# Characteristics and Outcomes of Pulmonary Embolism Patients Transferred After Activation of Pulmonary Embolism Response Team and Admitted from Local Emergency Department

**DOI:** 10.3390/jcm14030677

**Published:** 2025-01-22

**Authors:** Mateusz Jermakow, Michał Machowski, Magdalena Gałecka-Nowak, Karol Deutsch, Adam Il, Anna Imiela, Bartosz Karolak, Katarzyna Perzanowska-Brzeszkiewicz, Szymon Pacho, Agnieszka Wójcik, Marek Roik, Marcin Krakowian, Andrzej Łabyk, Marek Gołębiowski, Piotr Pruszczyk

**Affiliations:** 1 Department of Internal Medicine & Cardiology, Medical University of Warsaw, Lindleya 4 St., 02-005 Warsaw, Poland; 2 Department of Clinical Radiology, Medical University of Warsaw, Chałubińskiego 5 St., 02-004 Warsaw, Poland

**Keywords:** pulmonary embolism, pulmonary embolism response team, catheter-directed therapy, simplified pulmonary embolism severity index

## Abstract

**Background:** The primary role of a pulmonary embolism response team (PERT) is to support decision-making processes regarding acute pulmonary embolism (PE) patients and provide advanced rescue therapies when needed. Despite a great need for its availability among physicians, PERT’s usefulness is yet to be proven. **Objectives**: Our goal was to establish whether patients benefit from qualification by PERT for admission to a tertiary cardiology ward. **Methods**: Data of all patients hospitalized due to PE for 12 months (July 2023–June 2024) were retrospectively analyzed. We aimed to compare patients admitted primarily via the emergency department with those consulted by PERT and eventually transferred from other hospitals. The primary outcome was the use of advanced therapies. **Results:** We identified 167 patients (50.3% women) hospitalized with PE. Out of them, 102 (61.1%) came from the emergency department, while 65 (38.9%) patients were transferred after PERT consultation. The transferred patients generally had more severe conditions, as indicated by the ESC death risk group classification (intermediate-high and high risk, OR 19.2, 95% CI 8.3–44.2). They were more often qualified for at least one of the advanced therapies than the emergency department patients (OR 23.2, 95% CI 9.3–58.1). We found no significant differences in in-hospital mortality (6.9% versus 1.5%, OR 4.7, 95% CI 0.6–39.3). **Conclusions**: Establishing PERT as a reference unit providing advanced treatment resulted in successfully delivering more complex treatment to severely ill PE patients. Despite their unfavorable basic characteristics, neither length of hospitalization nor in-hospital mortality seem to differ when compared to unselected, less compromised cases.

## 1. Introduction

Acute pulmonary embolism (PE) remains the third most frequent acute cardiovascular disease, with an extremely heterogeneous clinical presentation [1]. The concept of the rapid evaluation of PE patients by a team of multidisciplinary professionals, known as the Pulmonary Embolism Response Team (PERT), was first implemented in 2013 in Massachusetts General Hospital in Boston (United States) [2]. Since then, it has gained growing recognition, both in the United States and in Europe [3].

In order to define and form the standards of PERT in Poland, the Polish PERT Initiative was established [4]. Initial experience suggested that PERTs provide a broad spectrum of consultations for hospitals not specialized in PE management and offer advanced therapies for patients with severe forms of PE transferred to a PERT center, though significant operational differences exist between PERT centers [5]. The central role of the PERT coordinator is to activate a team to elaborate the optimal patient management plan. Potential management recommendations include the continuation of anticoagulation at the referring site, the immediate start of the systemic thrombolysis (sTL) or the transfer of the patient to a PERT center if, in the consultant’s opinion, they may require advanced therapy.

Most trials assessed PERT’s efficacy by comparing patients managed within one center after establishing PERT to those managed in the pre-PERT era. Since forming a PERT in every hospital is virtually impossible, it seems crucial to implement a hub-and-spoke model and evaluate its efficacy.

Our objective in this retrospective study was to evaluate how PERT fulfills its role as a referral unit implementing novel management strategies. Therefore, we assessed the clinical course of patients managed in our center and compared the outcomes between a group of patients admitted from the hospital emergency department (ED) and a group of patients transferred from other centers.

## 2. Materials and Methods

All patients hospitalized in the referral hospital between July 2023 and June 2024 (12 months) with confirmed PE were included in the single-center retrospective study. They were at least 18 years old and had PE confirmed through a computed tomography pulmonary angiogram (CTPA).

Our referral hospital serves also as the primary medical unit for the local population. These patients are diagnosed in the ED and hospitalized afterwards. After admission, they are evaluated by PERT members according to the local protocol. Since even in the cases of the least severe patients, simple algorithms may be insufficient [6,7], all patients are consulted by experts in PE. Tailored decisions are made regarding optimal treatment, diagnosis, and further follow-up. This population of unselected cases of PE formed one study control group (ED-group).

The second population consisted of external patients transferred to our department only after PERT activation. According to the recommendations of the Polish PERT Initiative algorithm [4], a dedicated PERT consultant was available on a 24/7 basis to respond to calls from local health providers and to activate our PERT when indicated. Although PERT activity is not restricted to a specific area, it is usually activated by hospitals from its province. The basis of the consultation is the collection of all relevant data, mainly medical history, vital signs, CTPA description, and levels of myocardial injury biomarkers. Relying on gathered information, an initial patient’s profile is created in order to anticipate possible deterioration, either due to the progression of PE or because of serious bleeding complications. If the consultant decided on hospitalization, the patients were immediately transferred directly to the cardiology ward and managed according to PERT protocol.

Any implementation of advanced therapies is introduced after a thorough case review by PERT members. The core of the team is formed by clinicians directly involved in managing a patient, an echocardiographist, an interventional cardiologist, and a radiologist. If decided, experts from other fields are involved in the case work-up, mainly cardiac surgeons, pulmonologists, and anesthesiologists. The decision on starting any of advanced therapies and the choice of a specific method is made by consensus.

Clinical data of all patients were analyzed, including sPESI (simplified pulmonary embolism severity index) components, i.e., age, active cancer, chronic cardiopulmonary disease, heart rate, systolic blood pressure, and oxyhemoglobin saturation levels [8]. Besides sPESI score calculation, all patients had early mortality risk assessed as proposed by European Society of Cardiology (ESC) [1].

The primary outcome was the frequency of advanced therapy use, defined as sTL, referral for cardiac surgery, catheter-directed therapy (CDT), and vena cava filter (VCF) implantation. All-cause in-hospital mortality and PE-related mortality were also analyzed. The PE-related mortality comprised deaths caused by irreversible RV insufficiency or recurrent PE, based on clinical assessment. Moreover, the length of stay (LOS) in the hospital was collected, and in-hospitality mortality was recorded.

Since several randomized clinical trials testing the abovementioned therapies were being conducted in our center over the analyzed period of time, patients participating in them, but randomized to the control arm (i.e., anticoagulation-only therapy), were included in the advanced therapy group in our analysis. Primarily, they were found eligible for these therapies; secondly, the trials protocols implied more intense management than the standard of care.

Descriptive data are presented as “number (%)”, or as “median (interquartile range, IQR)” for continuous variables without normal distribution. Normality of distribution was verified with the Shapiro–Wilk test, skewness, and kurtosis values. Comparison of nonparametric values was made with the U Mann–Whitney test. Categorical variables were compared using the chi-square test or Fisher’s exact test. The alpha level of 0.05 was used for significance. Statistical analysis was performed using Statistica, version 13, TIBCO Software Inc. (2017, Palo Alto, CA, USA).

## 3. Results

**Figure 1:**
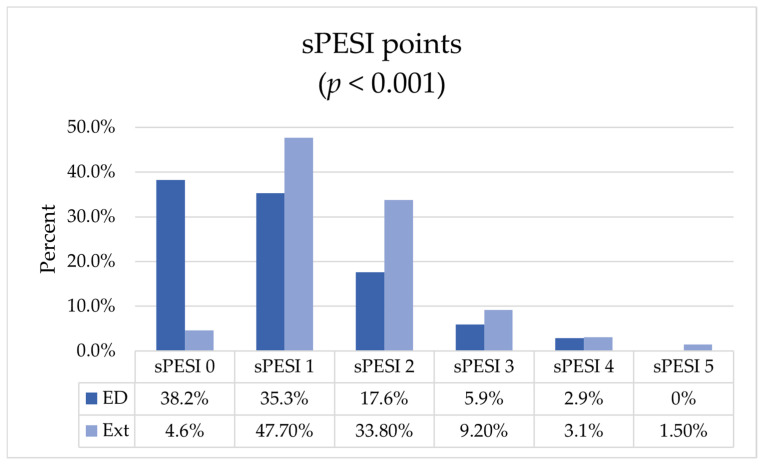
sPESI score distribution among patient groups. ED—emergency department, Ext—external patients; *p*-value calculated for U Mann–Whitney test.

We included consecutively 167 patients, 84 (50.3%) of whom were women, with a median age of 67 years (IQR 50–76), all with CTPA-confirmed PE. One hundred and two (61.1%) patients were primarily admitted to the ED, forming the control group (ED-group). The remaining 65 (38.9%) patients were admitted from other centers after PERT activation (the external group). The clinical characteristics of both groups are presented in Table 1 and in Figure 1.

There was an equal number of low and intermediate-low risk patients (42, 25.15%), but the most frequent risk group was intermediate-high (76, 45.5%). There were only 7 (4.2%) high-risk patients. These proportions, however, were not consistent between groups. Among the ED patients, the low-risk group was most frequently represented (39, 38.2%), followed by the intermediate-low group (38, 37.3%), while the majority of the external patients was in the intermediate-high group (52, 80%).

The median sPESI score in the whole group was 1 (IQR 0–2), with significantly different distribution among the ED patients (1, IQR 0–2) and the external patients (1, IQR 1–2). By excluding from the comparison the low-risk patients, who scored, by definition, 0 sPESI points and were overrepresented in the ED group, the difference vanished.

Chronic cardiopulmonary disease or cancer were identified in 40 (24%) and 29 (17.4%) patients, respectively. We did not find differences among groups in the proportion of patients with these comorbidities.

The majority of external patients (63.1%) qualified for some form of advanced treatment, while almost all patients from the ED-group (*n* = 95, 93.1%) received anticoagulation only (Table 2). One patient from each group of intermediate-low risk had VCF implanted. The remaining 46 patients undergoing any type of advanced treatment were either intermediate-high or high ESC risk.

The mean LOS was 6 days (4–7). In-hospital mortality was analyzed: 8 patients had died, resulting in an early mortality of 4.8%. A thorough analysis of causes of death was conducted. A death was deemed to be related to PE when the course of the patient’s hospitalization and subsequent deterioration was typical for gradual signs of right ventricle failure. Additionally, cases of sudden death without any better explanation were also considered to be PE-related. On the contrary, if death was preceded by cardiopulmonary stabilization, a severe terminal underlying disease coexisted, or PE seemed to be an incidental finding on CTPA during a routine work-up, the case wase considered to be unrelated to PE.

This analysis revealed that PE was the direct cause of death in two cases, resulting in an in-hospital PE-related mortality of 1.2% (Table 3). Detailed analysis of all fatal cases is presented in Appendix A Table A1.

## 4. Discussion

The establishment of PERT is encouraged by the ESC, although the exact composition and operating mode are not fixed [1]. A multicenter Polish analysis of four centers showed some significant differences in patients’ characteristics and their origins [5]. Nevertheless, a vast majority of physicians would like to consult a PE patient with PERT if presented with the possibility [9]. The main two reasons for a consultation were managing patients: (1) with severe symptoms (but not in shock) despite initial coagulation, or (2) with shock, but with contraindication for thrombolysis. One-third of physicians would consult a patient without shock nor right ventricle disfunction, but with massive PE on CTPA. The same study showed a need for increased guideline-recommended practices.

No prospective comparison of PERT implementation has been conducted. Available data so far have focused on comparing treatment before and after the establishment of PERT [3,10]. Despite their undoubted contribution, there are possibly some confounding factors resulting from the comparison of two consecutive periods of time. First, the availability of numerous therapies has improved over time and could have been inaccessible for patients in the pre-PERT era. Secondly, PERTs members managing patients might have been on different levels of a learning curve in the years preceding PERT’s implementation. Lastly, even good, up-to-date adherence to guidelines does not guarantee the homogeneity of any provided treatment, as the recommendations tend to be modified over time.

In our analysis, we focused on the utility of PERT compared to regular PE patients. Both groups were evaluated by the same team, whose core members included cardiologists, interventional cardiologists, internists, and radiologists. Decisions were made based on current guidelines and best clinical practice with regard to available therapeutic options at given moment. This approach additionally allowed the authors to exclude sampling bias, which could have resulted in favoring only selected patients.

The ED-group generally stays in line with previous cohorts [3,11]. The patients had a median age of 68 years (IQR 51–78), with equivalent sex distribution. There is a relatively large representation of low-risk patients. However, we counted all patients evaluated in ED, including those discharged home, which differs from other publications focused on only hospitalized patients.

In contrast, the external patients form a highly selected group. Consultations were initiated by the referring physician. If, in the dedicated PERT consultant’s opinion, a patient would benefit from advanced therapy, they were transferred directly to the cardiology ward. Our experience showed that physicians would most frequently seek PERT’s advice for challenging PE patients, who are at risk of decompensation or who cannot receive standard treatment because of contraindications [9].

In the external group, no differences regarding sex or age occurred. As anticipated, they were in a more severe condition, with 89.2% at intermediate-high or high risk of death, versus 24.5% in the ED-group (OR 19.2, 95% CI 8.3–44.2). The sPESI score was higher in the external group as well (Figure 1). This was mainly driven by clinical presentation factors, i.e., blood pressure, heart rate, and blood oxygenation, as no significant differences were shown regarding chronic cardiopulmonary diseases or active cancer. When excluding low-risk patients from the analysis, e.g., those discharged early or not admitted at all, no significant differences in sPESI score between groups were found.

External patients were more often treated with advanced methods, including catheter-directed therapies, cardiac surgery, or VCF implantation. Only three patients with low-risk PE and contraindications for anticoagulation were admitted for VCF implantation. Of note, only a few cases were treated with primary sTL, therefore we were not able to show significant differences in the use of it. This reflects the previously observed progressive shift towards CDT [12,13,14]. Over the analyzed period, only two interventional clinical trials controlled with anticoagulation were conducted [15,16]. Since some patients were found eligible for either sTL or CDT, inclusion in a clinical trial was also considered as using an advanced method of PE treatment. In general, there was a significant difference in the odds of receiving at least one form of advanced therapys (OR 23.2, 95% CI 9.3–58.1).

This disproportion results from a relatively large group of non-severely ill patients in the ED-group. Nonetheless, even after accounting for only intermediate-high and high-risk patients, the external group was treated more aggressively (69% versus 24%; OR 7.0, 95% CI 2.4–20.6). This suggests that additional factors than simple ESC risk stratification or sPESI score were considered in the decision-making process. Other elements have proven to be more useful in predicting medium-term mortality in PE patients [17].

The in-hospital mortality was low, with 8 deaths (4.8% of all patients) in total and only one in each group directly caused by PE, which is similar to the rates observed in PERT registries [5]. The majority of deaths were caused by severe comorbidities, including advanced cancer. Additionally, in both cases of death due to PE, there were also severe comorbidities present.

Since the number of deaths was low in both groups, either overall or regarding ESC-risk sub-groups, we were not able to analyze with sufficient credibility the observed differences in mortality. However, the percentage of deaths for the external group is at least not worse than in the unselected ED-group.

We did not find significant differences in LOS between the ED and external groups. However, it should be noted that the external group comprised patients with more severe forms of PE. The shortening of LOS after PERT implementation [3] seems to affect PE patients equivalently, regardless of risk group, when managed by PERT.

There are some apparent limitations. First, this is a retrospective observational study from a single center. We have not recorded all the PERT activations, so the characteristics of patients not eligible for admission are unknown. Consequently, our conclusions are limited to those eventually hospitalized in the referral unit. This may result in a loss of information about patients who received sTL on site, were transferred directly to cardiac surgery hospital, or were disqualified from transfer either because of severe condition or terminal comorbidities.

Significant differences in advanced treatment cannot be explained solely by ESC risk stratification or sPESI score. Supposedly, there were other clinical factors that implicated such prominent differences. In fact, appropriate algorithms for identifying and managing patients at risk of deterioration are still a matter of discussion [13], and our study was not aimed at nor sufficiently powered to identify these factors.

There was also a moderate loss of follow-up for 8.4% of all patients, mainly in the ED-group. A majority of these patients had advanced cancer and were referred to palliative care. As the 30-day survival is unknown for these patients, the general mortality rate may be bigger than observed.

## 5. Conclusions

The role of PERT in managing therapeutically challenging PE patients seems to be fundamental. Creating a network of hospitals with hub units located in experienced centers allows for the selection of patients requiring advanced treatment and the delivery of such treatment when needed. Despite recruiting more severely ill patients, a low PE-related mortality was observed, and no prolongation of hospital stay was found.

## Figures and Tables

**Table 1:** Clinical characteristics of patients with acute PE.

Parameter	All Patients (*n* = 167)	ED (*n* = 102)	External (*n* = 65)	*p*-Value External vs. ED
Sex F/M (*n*, %F)	84/83 (50.3%)	55/47 (53.9%)	29/36 (44.6%)	ns.
Median age (IQR)	67 (50–76)	68 (51–78)	66 (49–74)	ns.
ESC risk Low Int-Low Int-High High	42 (25.15%) 42 (25.15%) 76 (45.5%) 7 (4.2%)	39 (38.2%) 38 (37.1%) 24 (23.5%) 1 (1.0%)	3 (4.6%) 4 (6.2%) 52 (80%) 6 (9.2%)	*p* < 0.001
sPESI index all patients not-low risk patients	1 (0–2) 1 (1–2)	1 (0–2) 1 (1–2)	1 (1–2) 1.5 (1–2)	*p* < 0.001 ns.
Comorbidities: Chronic cardiopulmonary disease Cancer	40 (24.0%) 29 (17.4%)	26 (25.5%) 22 (21.6%)	14 (21.5%) 7 (10.8%)	ns. ns.

Calculated for intermediate-risk patients only; ED—emergency department, ns.—not significant.

**Table 2:** Methods of advanced treatment.

Treatment	All Patients (*n* = 167)	ED (*n* = 102)	External (*n* = 65)	*p*-Value External vs. ED
Anticoagulation only	119 (71.3%)	95 (93.1%)	24 (36.9%)	*p* < 0.001
Any form of advanced therapy	48 (28.7%)	7 (6.9%)	41 (63.1%)	
Systemic thrombolysis	3 (1.8%)	0 (0.0%)	3 (4.5%)	ns.
Cardiac surgery	5 (3.0%)	0 (0.0%)	5 (7.5%)	*p* < 0.05
Vena cava filter placement	13 (7.8%)	2 (2.0%)	11 (16.4%)	*p* < 0.01
Catheter-directed therapy	23 (13.8%)	2 (2.0%)	21 (31.3%)	*p* < 0.001
Interventional clinical trial	25 (15.0%)	4 (3.9%)	21 (31.3%)	*p* < 0.001

ED—emergency department, ns.—not significant.

**Table 3:** Length of stay and in-hospital mortality.

	All Patients (*n* = 167)	ED (*n* = 102)	External (*n* = 65)	*p*-Value External vs. ED
LOS, days (IQR) -total -ESC intermediate-high and high	6 (4–7) 6 (5–8)	3 (3–7.5) 6.5 (5–9)	6 (5–7) 6 (5–7)	ns. ns.
In-hospital all-cause mortality, *n* (%) -total, *n* (%) ESC risk -low -int-low int-high -high	8 (4.8%) 0 3 3 2	7 (6.9%) 0 3 3 1	1 (1.5%) 0 0 0 1	ns. nc.
PE-related in-hospital mortality, *n* (%)	2 (1.2%)	2	0	nc.

ED—emergency department, nc.—not calculated, ns.—not significant.

## Data Availability

Data are available from the corresponding author upon written request.

## Abbreviations

The following abbreviations are used in this manuscript:

CDT	catheter-directed therapy
CTPA	computed tomography pulmonary angiogram
ED	emergency department
ESC	European Society of Cardiology
IQR	interquartile range
LOS	length of stay
PE	pulmonary embolism
PERT	pulmonary embolism response team
sPESI	simplified pulmonary embolism severity index
sTL	systemic thrombolysis
VCF	vena cava filter

## Appendix A

**Table A1:** In-hospital deaths details.

Age	ESC Risk	Origin	sPESI	Comorbidities, Course of Hospitalization	Death Related to PE	Time to Death from Diagnosis (Days)
77	Int-high	ED	2	Chronic interstitial advanced lung pneumonia, home oxygen therapy. In CTPA radiologically massive pulmonary embolism.	Yes	3
87	Int-high	ED	3	Femur fracture. After cardiopulmonary stabilization, and VCF implantation transferred to orthopedic ward without right ventricular dysfunction. Sudden death two days post operation, recurrent PE cannot be excluded.	Yes	8
74	High	Ext	3	Chronic alcohol abuse, malnutrition. Cardiac arrest. Stabilized with CDT intervention. Deteriorated after 5 days due to ischemic stroke. Death caused by ventilator-associated pneumonia.	No	15
77	Int-high	ED	3	Chronic obstructive pulmonary disease with emphysema and secondary pulmonary hypertension. Malnutrition. Home oxygen therapy. Only segmental emboli on CTPA.	No	9
60	Int-low	ED	3	Metastatic colon cancer on palliative chemotherapy. Pulmonary embolism accidentally found on CTPA for staging. Died due to septic shock.	No	9
78	High	ED	4	Chronic alcohol abuse, malnutrition, found hypothermic. Died due to septic shock.	No	18
76	Int-low	ED	2	Metastatic pancreatic cancer. Terminally ill.	No	5
68	Int-low	ED	3	Metastatic bile duct cancer. Pneumonia. Terminally ill.	No	10

ED—emergency department, Ext—external patients.

## References

[1] Konstantinides S.V., Meyer G., Becattini C., Bueno H., Geersing G.-J., Harjola V.-P., Huisman M.V., Humbert M., Jennings C.S., Jiménez D. (2019). 2019 ESC Guidelines for the diagnosis and management of acute pulmonary embolism developed in collaboration with the European Respiratory Society (ERS). Eur. Hear. J..

[2] Provias T., Dudzinski D.M., Jaff M.R., Rosenfield K., Channick R., Baker J., Weinberg I., Donaldson C., Narayan R., Rassi A.N. (2014). The Massachusetts General Hospital Pulmonary Embolism Response Team (MGH PERT): Creation of a Multidisciplinary Program to Improve Care of Patients With Massive and Submassive Pulmonary Embolism. Hosp. Pr..

[3] Hobohm L., Farmakis I.T., Keller K., Scibior B., Mavromanoli A.C., Sagoschen I., Münzel T., Ahrens I., Konstantinides S. (2022). Pulmonary embolism response team (PERT) implementation and its clinical value across countries: A scoping review and meta-analysis. Clin. Res. Cardiol..

[4] Araszkiewicz A., Kurzyna M., Kopeć G., Roik M., Darocha S., Pietrasik A., Puślecki M., Biederman A., Przybylski R., Stępniewski J. (2020). Expert opinion on the creating and operating of the regional Pulmonary Embolism Response Teams (PERT). Polish PERT Initiative. Cardiol. J..

[5] Araszkiewicz A., Kurzyna M., Kopeć G., Sławek-Szmyt S., Wrona K., Stępniewski J., Jankiewicz S., Pietrasik A., Machowski M., Darocha S. (2021). Pulmonary embolism response team: A multidisciplinary approach to pulmonary embolism treatment. Polish PERT Initiative Report. Kardiologia Polska.

[6] Karolak B., Ciurzyński M., Skowrońska M., Kurnicka K., Pływaczewska M., Furdyna A., Perzanowska-Brzeszkiewicz K., Lichodziejewska B., Pacho S., Machowski M. (2023). Plasma Troponins Identify Patients with Very Low-Risk Acute Pulmonary Embolism. J. Clin. Med..

[7] Karolak B., Skowrońska M., Machowski M., Dzikowska-Diduch O., Bienias P., Kuryła M., Wiśniewska M., Gołębiowski M., Pruszczyk P., Ciurzyński M. (2024). Plasma N-terminal pro-brain natriuretic peptide concentrations may help to identify patients with very low-risk acute pulmonary embolism: A preliminary study. Adv. Clin. Exp. Med..

[8] Jiménez D., Aujesky D., Moores L., Gómez V., Lobo J.L., Uresandi F., Otero R., Monreal M., Muriel A., Yusen R.D. (2010). Simplification of the Pulmonary Embolism Severity Index for Prognostication in Patients With Acute Symptomatic Pulmonary Embolism. Arch. Intern. Med..

[9] Stępniewski J., Wilczek Ł., Waligóra M., Jonas K., Magoń W., Furtak R., Curzytek A., Kurzyna M., Araszkiewicz A., Frołow M. (2024). Current practices in diagnosing acute pulmonary embolism: A comprehensive analysis of adherence to contemporary practice guidelines. Pol Arch Intern Med..

[10] Sagoschen I., Scibior B., Farmakis I.T., Keller K., Graafen D., Griemert E.-V., Vosseler M., Treede H., Münzel T., Knorr M. (2023). A multidisciplinary pulmonary embolism response team (PERT): First experience from a single center in Germany. Clin. Res. Cardiol..

[11] Schultz J., Giordano N., Zheng H., Parry B.A., Barnes G.D., Heresi G.A., Jaber W., Wood T., Todoran T., Courtney D.M. (2019). EXPRESS: A Multidisciplinary Pulmonary Embolism Response Team (PERT)—Experience from a national multicenter consortium. Pulm Circ..

[12] Rosovsky R., Chang Y., Rosenfield K., Channick R., Jaff M.R., Weinberg I., Sundt T., Witkin A., Rodriguez-Lopez J., Parry B.A. (2018). Changes in treatment and outcomes after creation of a pulmonary embolism response team (PERT), a 10-year analysis. J. Thromb. Thrombolysis.

[13] Pruszczyk P., Klok F.K., Kucher N., Roik M., Meneveau N., Sharp A.S., Nielsen-Kudsk J.N.-K., Obradović S., Barco S., Giannini F. (2022). Percutaneous treatment options for acute pulmonary embolism: A clinical consensus statement by the ESC Working Group on Pulmonary Circulation and Right Ventricular Function and the European Association of Percutaneous Cardiovascular Interventions. EuroIntervention.

[14] Kopeć G., Araszkiewicz A., Kurzyna M., Sławek-Szmyt S., Stępniewski J., Roik M., Darocha S., Gołębiowski M., Jaguszewski M., Jankiewicz S. (2023). Role of catheter-directed therapies in the treatment of acute pulmonary embolism. Expert opinion of the Polish PERT Initiative, Working Group on Pulmonary Circulation, Association of Cardiovascular Interventions, and Association of Intensive Cardiac Care of the Polish Cardiac Society. Kardiologia Polska.

[15] Klok F.A., Piazza G., Sharp A.S., Ainle F.N., Jaff M.R., Chauhan N., Patel B., Barco S., Goldhaber S.Z., Kucher N. (2022). Ultrasound-facilitated, catheter-directed thrombolysis vs anticoagulation alone for acute intermediate-high-risk pulmonary embolism: Rationale and design of the HI-PEITHO study. Am. Hear. J..

[16] Sanchez O., Charles-Nelson A., Ageno W., Barco S., Binder H., Chatellier G., Duerschmied D., Empen K., Ferreira M., Girard P. (2021). Reduced-Dose Intravenous Thrombolysis for Acute Intermediate–High-risk Pulmonary Embolism: Rationale and Design of the Pulmonary Embolism International THrOmbolysis (PEITHO)-3 trial. Thromb. Haemost..

[17] Imiela A.M., Kozak-Szkopek E., Szymańska M., Wdowiak K., Dzikowska-Diduch O., Żuk-Łapan A., Pruszczyk A., Bukalska I., Niewczas K., Machowski M. (2024). The Vulnerable Elders Survey-13 scale is superior to the simplified Pulmonary Embolism Score Index in predicting 3-month postdischarge mortality in elderly survivors of acute pulmonary embolism. Pol Arch Intern Med..

